# Perinatal Photoperiod Associations With Allergic and Respiratory Disease in the UK Biobank Database

**DOI:** 10.1111/all.16508

**Published:** 2025-02-20

**Authors:** Jonas P. Wallraff, Achim Traub, Peter Morfeld, Martin Hellmich, Thomas C. Erren, Philip Lewis

**Affiliations:** ^1^ Institute and Policlinic for Occupational Medicine, Environmental Medicine and Prevention Research, Faculty of Medicine and University Hospital of Cologne University of Cologne Cologne Germany; ^2^ Institute of Medical Statistics and Computational Biology, Medical Faculty and University Hospital of Cologne University of Cologne Cologne Germany

**Keywords:** circadian, light, perinatal, photoperiod, PLICCS

To the Editor,

1

Allergy/respiratory diseases affecting millions worldwide pose a substantial health and economic burden. Experimental research, supported by epidemiological studies of season of birth, indicates early life factors affect later life allergy/respiratory disease risk. Perinatal light imprinting circadian clocks and systems (PLICCS) may affect later life disease risk as animals exposed to different perinatal 24‐h light/dark paradigms present with different circadian biology, physiology and behavioural responses [1]. PLICCS is supported by epidemiological associations between perinatal natural photoperiod (daylight hours from sunrise to sunset) and risk of cancer, diabetes, depression and bipolar disorder [2, 3, 4, 5, 6]. Circadian biology co‐governs immune function. Thus, perinatal photoperiod may affect later life risk of allergy/respiratory diseases (see Data S1 for further introduction).

Herein, we report cross‐sectional associations between perinatal natural photoperiod metrics and the prevalence of asthma, hay fever/allergic rhinitis/eczema (HAE), and COPD in 451,552 UK Biobank participants. The metrics include mean daily photoperiod and photoperiod range relative to the mean in the 3rd trimester and 3 months post‐birth time windows. Asthma, HAE and COPD outcomes were determined by questionnaire. Multivariable logistic regression (Table S1) was used to determine odds ratios (ORs) and 95% confidence intervals (95%CIs).

Generally, photoperiod metrics were differentially associated with changes in odds of all outcomes and differentially by groups of more or less extreme perinatal photoperiod exposures (Tables 1 and 2). For participants who experienced photoperiods between 8 and 16 h, 3rd trimester relative photoperiod range associations with asthma, HAE and COPD are OR 1.49 (95% CI 1.22–1.80), OR 0.95 (95% CI 0.82–1.09) and OR 2.66 (95% CI 1.46–4.84), respectively. We expect that participants will avoid light beyond 16 h per day in order to sleep and will seek artificial light when daylight is < 8 h (that may affect circadian biology when there is less daylight). Thus, focus on those that experienced only photoperiods between 8 and 16 h is important. We consider model 2 in the tables to be the most reliable (additional covariates in model 3 may have changed post diagnoses). Statistically significant results are also observed for participants who experienced more extreme photoperiods (Tables 1 and 2).

**TABLE 1:** Odds ratios & corresponding 95% intervals for 3rd trimester photoperiod metric associations with allergies and respiratory diseases in the UK Biobank.

	Mean daily photoperiod			Relative photoperiod range		
	Model 1	Model 2	Model 3	Model 1	Model 2	Model 3
Asthma						
ESP	1.09 (1.06–1.13)	1.09 (1.05–1.12)	1.05 (1.01–1.07)	0.50 (0.40–0.63)	0.52 (0.41–0.65)	0.72 (0.55–0.96)
NEP	0.99 (0.97–1.02)	1.00 (0.98–1.02)	1.01 (0.98–1.03)	1.25 (1.10–1.44)	1.49 (1.22–1.80)	1.71 (1.38–2.12)
ELP*	0.85 (0.82–0.88)	0.86 (0.83–0.89)	0.95 (0.90–1.00)	0.20 (0.14–0.29)	0.22 (0.15–0.32)	0.52 (0.32–0.84)
Hay fever, allergic rhinitis or eczema (HAE)						
ESP	1.14 (1.11–1.17)	1.14 (1.12–1.16)	1.05 (1.02–1.08)	0.32 (0.27–0.37)	0.33 (0.28–0.39)	0.64 (0.52–0.79)
NEP	0.98 (0.96–1.00)	0.99 (0.97–1.00)	1.00 (0.98–1.02)	0.76 (0.69–0.83)	0.95 (0.82–1.09)	1.22 (1.05–1.43)
ELP*	0.80 (0.78–0.83)	0.81 (0.78–0.83)	0.92 (0.88–0.96)	0.13 (0.10–0.17)	0.14 (0.11–0.18)	0.44 (0.31–0.63)
Emphysema & chronic bronchitis (COPD)						
ESP	0.87 (0.81–0.93)	0.87 (0.81–0.94)	0.96 (0.88–1.05)	3.19 (1.89–5.41)	3.10 (1.82–5.26)	1.30 (0.66–2.60)
NEP	1.03 (0.97–1.10)	1.02 (0.96–1.09)	0.99 (0.93–1.06)	5.01 (3.26–7.69)	2.66 (1.46–4.84)	1.80 (0.92–3.51)
ELP*	1.07 (0.98–1.18)	1.06 (0.97–1.17)	0.92 (0.81–1.05)	2.14 (0.90–5.08)	1.99 (0.83–4.75)	0.50 (0.15–1.72)

*Note:* Model 1: photoperiod metrics only; Model 2: model 1 + sex, ethnicity, age; Model 3: model 2 + chronotype, Townsend deprivation index, education, test centre; Perinatal Photoperiod Metrics: Mean daily photoperiod = average of photoperiods in the 3rd trimester; Photoperiod relative range = difference between longest and shortest photoperiods in the 3rd trimester relative to the mean; Photoperiod Groups (by photoperiods experienced in the 3rd trimester): ESP = extreme short photoperiods (individuals who experience at least 1 day with photoperiod < 8 h); NEP = non‐extreme photoperiod (individuals who experience 3rd trimester photoperiods between 8 and 16 h exclusively); ELP = extreme long photoperiods (individuals who experience at least 1 day with photoperiod > 16 h).

* Multicollinearity was observed in the ELP groups; see Supplementary Discussion in Data S1 for consideration.

**TABLE 2:** Odds ratios & corresponding 95% intervals for 3 months post‐birth photoperiod metric associations with allergies and respiratory diseases in the UK Biobank.

	Mean daily photoperiod			Relative photoperiod range		
	Model 1	Model 2	Model 3	Model 1	Model 2	Model 3
Asthma						
ESP	1.07 (1.03–1.10)	1.07 (1.03–1.10)	1.02 (0.98–1.06)	0.49 (0.39–0.62)	0.51 (0.40–0.64)	0.76 (0.57–1.01)
NEP	0.98 (0.96–1.00)	0.98 (0.96–1.01)	0.99 (0.96–1.01)	1.21 (1.05–1.38)	1.38 (1.13–1.67)	1.53 (1.23–1.89)
ELP*	0.86 (0.83–0.89)	0.87 (0.84–0.90)	0.93 (0.88–0.98)	0.23 (0.16–0.33)	0.25 (0.18–0.34)	0.56 (0.29–0.73)
Hay fever, allergic rhinitis or eczema (HAE)						
ESP	1.13 (1.10–1.16)	1.13 (1.10–1.15)	1.04 (1.01–1.07)	0.40 (0.33–0.47)	0.41 (0.34–0.49)	0.80 (0.64–1.00)
NEP	1.00 (0.98–1.02)	1.00 (0.98–1.02)	1.01 (0.99–1.03)	0.76 (0.69–0.84)	0.91 (0.79–1.05)	1.20 (1.03–1.41)
ELP*	0.80 (0.78–0.83)	0.81 (0.79–0.83)	0.92 (0.89–0.96)	0.11 (0.08–0.14)	0.12 (0.90–0.15)	0.40 (0.28–0.57)
Emphysema & chronic bronchitis (COPD)						
ESP	0.82 (0.76–0.89)	0.82 (0.76–0.88)	0.89 (0.81–0.98)	3.11 (1.82–5.32)	3.08 (1.78–5.29)	1.57 (0.77–3.19)
NEP	1.08 (1.02–1.15)	1.07 (1.01–1.14)	1.07 (1.00–1.15)	5.09 (3.30–7.86)	3.42 (1.85–6.30)	2.92 (1.46–5.86)
ELP*	1.13 (1.04–1.24)	1.26 (1.03–1.23)	0.96 (0.85–1.09)	3.00 (1.30–6.94)	2.86 (1.23–6.66)	0.63 (0.19–2.08)

*Note:* Model 1: photoperiod metrics only; Model 2: model 1 + sex, ethnicity and age; Model 3: model 2 + chronotype, Townsend deprivation index, education, test centre; Perinatal Photoperiod Metrics: Mean daily photoperiod = average of photoperiods in the 3 months post‐birth; Photoperiod relative range = difference between longest and shortest photoperiods in the 3 months post‐birth relative to the mean; Photoperiod Groups (by photoperiods experienced in the 3 months post‐birth): ESP = extreme short photoperiods (individuals who experience at least 1 day with photoperiod < 8 h); NEP = non‐extreme photoperiod (individuals who experience 3rd trimester photoperiods between 8 and 16 h exclusively); ELP = extreme long photoperiods (individuals who experience at least 1 day with photoperiod > 16 h).

* Multicollinearity was observed in the ELP groups; see Supplementary Discussion in Data S1 for consideration.

These findings align with the PLICCS hypothesis [1]. Consistency (for the most part) across the three models, between perinatal time windows, and similar results for asthma and HAE lend support to validity; the latter also suggests a similar mechanism (not unexpected as they can both be categorised as allergic disorders).

Given strong links between smoking and COPD, we also stratified by ‘ever smoker yes/no’ and assessed a photoperiod range × pack years interaction term with COPD as outcome. Only some weak indications of effect modification are observed (Table S2). However, as early life exposures may prime the lungs to susceptibility to damage, it would be remiss to rule out potential effect modification at this stage.

A healthy volunteer bias and cross‐sectional design disallow disentangling longitudinal associations of covariates with incidence data (necessary to identify causal associations) and may lead to spurious associations. There may also be residual confounding by other potential risk factors that are differentially distributed by exposure metrics. However, as a first exploration of associations between perinatal photoperiods and allergy/respiratory diseases, with a large sample size and number of cases, our findings suggest that further exploration is warranted. Elaboration upon methods and discussion can be found in the Data S1.

In conclusion, PLICCS may reconcile inconsistencies in season‐of‐birth studies and represent a way to reduce the risk of allergy/respiratory disease.

## Author Contributions

P.L. and T.C.E. conceived the project. J.P.W. conducted the analyses and wrote the first draft. P.M. and M.H. contributed statistical expertise. A.T. conducted pertinent background research. All authors provided input to and approved of the final draft for submission and publication.

## Conflicts of Interest

The authors declare no conflicts of interest.

## Supporting information


Data S1.

## Data Availability

Data requests should be directed to the UK Biobank.
