# Supplementary material for: Perinatal Photoperiod Associations With Allergic and Respiratory Disease in the UK Biobank Database
Source: Allergy. 2025 Feb 20;80(7):2047–9. doi: 10.1111/all.16508 (PMC12261865; doi:10.1111/all.16508)
Supplement: Supplementary file 1 — Data S1. [file ALL-80-2047-s001.docx]

**Perinatal Photoperiod Associations with Allergic & Respiratory Disease in the UK Biobank Database**

**Supplementary Introduction**

Allergic inflammatory diseases are heavily influenced by type 2 immune responses orchestrated by type 2 t-helper cells (T_H_2) and cytokines secreted by type 2 innate lymphoid cells (ILC2).^1–3^ ILC2s also contribute to the immune response associated with T_H_2 in chronic obstructive pulmonary disease (COPD).^4^ During foetal development, the T_H_2 immune response predominates with the T_H_1 response believed to be suppressed.^1,3,5^ This gradually shifts towards T_H_1 dominance at birth with dendritic cell transition to T_H_1 dominance during the 3^rd^ trimester.^3^ This shift is vulnerable to disruption by early life environmental factors.^1,3,5,6^ Spring/summer birth has been associated with a predominant T_H_2-related cytokine response in cord blood.^7,8^ The transcription factors CLOCK and BMAL1 promote expression of core ‘clock’ genes with nuclear receptors (ROR) forming another regulatory loop.^9^ In mice, RORα regulates the transcription of the core ‘clock’ gene *Bmal1*, which may play a role in asthma development.^10,11^ Furthermore, RORα is essential for the development of ILC2 cells involved in type 2 inflammation and appears to govern the T_H_2-cell response.^12–14^ Hence, the distinctive function of RORα implies that allergic responses are regulated by the CTS.^15^ ‘Clock’ genes have also been implicated in pathogenesis and progression of obstructive lung disease.^16,17^ Season and latitude of birth – determinants of photoperiod (hours of daylight from sunrise to sunset) – have also both been associated with epigenetic remodelling.^18^ Circadian timing systems co-govern immune system function.^19^ Taken together (alongside main text material), perinatal photoperiod links PLICCS and circadian biology with immune system function and allergic and respiratory disease, with PLICCS as a mechanistic hypothesis. As asthma, allergic rhinitis and COPD pose substantial socioeconomic burden by affecting millions worldwide,^20–24^ perinatal photoperiod warrants investigation as a potential early life risk factor.

**Supplementary Methods**

Approval was granted by the UK Biobank (Project 44219) and the Ethics Commission of the Medical Faculty of the University of Cologne. The study was conducted in accordance with the principles of the Declaration of Helsinki.^25^ The UK Biobank project is conducted with informed consent. The UK Biobank recruited ~0.5 million volunteers between 2006 and 2010 by postal invitation (invitations were sent to ~9 million individuals, aged 38–70 years, and residing within ~40 km of UK Biobank assessment centres across most of the UK).^26,27^ As participants were found less likely to be obese, smoke, consume alcohol daily, and reported fewer health conditions compared to the general population, there may be a healthy volunteer bias.^28^ Nonetheless, prevalence of hay fever/allergic rhinitis and COPD is in accordance with the reported UK adult prevalences (~26% and ~2%, respectively).^29,30^ The prevalence of asthma (~20%) is slightly higher than in UK Biobank respondents.^31^

A solar calculator provided natural photoperiod information for each day of the year at each latitude (<https://gml.noaa.gov/grad/solcalc/>). Using individual time-of-year of birth and birth location data, we assigned individual mean daily photoperiod and photoperiod relative range metrics for perinatal time windows of interest (namely, the 3^rd^ trimester and 3 months post birth; see previous publications for further details). Mean daily photoperiod is the average of photoperiods in the time window of interest. Photoperiod relative range is the difference between the longest and shortest photoperiods in the time window of interest relative to the mean. The metrics are associated in an inverted U-shaped fashion.^32^ We have previously rationalised that regression analyses may depend on joint population distribution of individual central tendency and dispersion descriptors of perinatal photoperiod in the time windows of interest.^32^ We grouped study participants according to whether or not they experienced extreme photoperiods or not during the time windows of interest. We define one group that experienced at least one day < 8 hours photoperiod (ESP – extreme short photoperiod) in the perinatal time window of interest, one group with at least one day > 16 hours photoperiod (ELP – extreme long photoperiod), and the rest as the non-extreme photoperiod (NEP) group. We expect that participants will avoid light beyond 16 hours per day in order to sleep and will seek artificial light that may affect circadian timing systems when daylight is <8 hours; thus, the NEP group is particularly important. In addition, more extreme perinatal photoperiods are identified as important from studies in animal models.^33^ Participants self-reported whether they had ever been diagnosed by a doctor with any of asthma, HAE, and COPD. Townsend deprivation index (TDI) is based on national census data and participant postcode. Education is based on the national qualifications framework in the UK. Chronotype is determined by asking if participants consider themselves either a definite morning person, more morning than evening, more evening than morning, definite evening person, or unknown/prefer not to answer.

We used multivariable logistic regression to evaluate associations between perinatal photoperiod metrics and prevalences of asthma, HAE, and COPD. Perinatal photoperiod metrics were entered simultaneously into statistical model 1. In model 2, we add non-modifiable covariates to model 1. In model 3, we also add modifiable covariates (chronotype, TDI, education, and test centre) that may have changed post diagnoses to model 2; however, “ever smoker - yes/no” and pack-years were not used at this stage due to potentially non-negligible missing data. Chronotype was included as a potential confounder as it has been associated with perinatal photoperiod metrics^32^ and allergic disorders and COPD.^34–38^ Since we lack data on chronotype pre-diagnoses and only have information from the start of the cross-sectional study, we did not investigate potential effect modifications by chronotype. Note, test centre proxies location at the time of recruitment and is not equivalent to perinatal location. For longitudinal studies, recommendations outlined by Erren & Morfeld (2024) can be followed.^39^ Considering that smoking is a strong predictor of COPD and due to missing data, we stratified by “ever smoker - yes/no” and conducted model 2 regression analyses of the associations between perinatal photoperiod and COPD. Lastly, in the “ever smoker - yes” stratum, we assessed inclusion of pack years as a continuous covariate and pack years × relative photoperiod range interaction terms. We note potential issues surrounding temporality regarding the modifiable covariates (including test-centre) and disease in this cross-sectional study. Likelihood ratio tests were used to compare nested models. Multicollinearity was assessed by variance inflation factors (VIFs). Odds ratios (ORs) and 95% confidence intervals (95% CIs) are reported. All analyses were conducted with Stata, Version 15.1 (Stata-Corp LLC, College Station, TX, USA). We did not adjust for multiplicity to guard against loss of statistical power – instead, we discuss the risk of detecting spurious associations as a limitation.^40^

**Supplementary Discussion**

The relative photoperiod range metric warrants further discussion and context. Let us compare hypothetical birth scenarios in the very south (London, 51°N) and north of the UK (Aberdeen, 57°N) on different dates throughout the year. For instance, comparing a winter birth in London vs Aberdeen, the 3^rd^ trimester relative photoperiod range differs by 0.16 (determined using data from <https://gml.noaa.gov/grad/solcalc/>) because of latitude. Comparing late summer births with early summer births in Aberdeen, 3^rd^ trimester relative photoperiod range can differ by 0.24 because of time-of-year. Thus, a 1-unit change in relative photoperiod range is considerable in comparison to mean daily photoperiod across ~3 months of the year (mean daily photoperiod can differ by 8 hours in Aberdeen alone). Nonetheless, differences in relative photoperiod range because of time-of-year and latitude such as in the example (e.g., fractions of unit) would still translate into conspicuous relative effect sizes for these highly prevalent respiratory and allergic disorders.

Multicollinearity was observed in the ELP groups. The source was the exposure metrics in this group, identified by leaving one out in turn. Leaving one metric out resulted in the mean metric estimates for asthma and HAE changing from statistically significant to not statistically significant. Other estimates remain unchanged regarding statistical significance and direction of effect, but with lower effect size. Even so, our rational is that both metrics can be important in the models. Overall, with our focus on the NEP group as most important, this does not change our conclusions.

Additionally, regarding limitations, temporality is an issue for the modifiable covariates in model 3 as these may have changed post disease diagnosis, including test centre. There may still be residual confounding by other potential risk factors that are differentially distributed by perinatal photoperiod metrics (e.g., socioeconomic status)^41^ and by those that cannot be well assessed in cross-sectional analyses such as pack years. The distribution of other environmental factors across both time-of-year and latitude are not as predictable as photoperiod, but we cannot rule them out as potential sources of some residual confounding.

**Table S1: Descriptive data**

|  |  | **Asthma^†^** | **HAE^†1^** | **COPD^†2^** | **Non-cases** |
| --- | --- | --- | --- | --- | --- |
| **Count** |  | 53,408 | 106,642 | 7,653 | 311,657 |
| Female | (%) | 30,944 (57.94) | 62,539 (58.64) | 3,900 (50.96) | 164,142 (52.67) |
| **Age** | Mean (SD) | 53.41 (8.25) | 55.11 (8.11) | 60.21 (6.89) | 56.96 (7.98) |
|  | Range | 38-70 | 39-72 | 40-70 | 39-73 |
| **Ethnicity** | White (%) | 51,424 (96.60) | 102,357 (96.26) | 7,491 (98.28) | 301,719 (96.81) |
|  | Non-White (%) | 1,808 (3.40) | 3,973 (3.74) | 131 (1.72) | 9,053 (3.19) |
|  | Unknown/Missing (%) | 176 (0.33) | 312 (0.30) | 31 (0.41) | 885 (0.28) |
| **Chronotype** | Definitely Morning (%) | 12,780 (26.87) | 22,898 (24.13) | 1,847 (27.13) | 75,624 (27.39) |
|  | Morning > Evening (%) | 16,082 (33.81) | 33,727 (35.54) | 1,937 (28.46) | 99,847 (36.16) |
|  | Evening > Morning (%) | 13,645 (28.69) | 28,308 (29.83) | 2,140 (31.44) | 77,740 (28.16) |
|  | Definitely Evening (%) | 5,055 (10.63) | 9,972 (10.51) | 883 (12.97) | 22,884 (8.29) |
|  | Unknown/Missing (%) | 5,846 (10.95) | 11,377 (10.67) | 846 (11.05) | 35,562 (11.41) |
| **TDI^3^** | Mean (SD) | -1.18 (3.16) | -1.51 (2.97) | 0.01 (3.53) | -1.44 (3.01) |
|  | Unknown/Missing (%) | 86 (0.16) | 163 (0.15) | 11 (0.14) | 427 (0.14) |
| **Qualification^‡^** | NQF7+ (%) | 25,197 (49.34) | 57,241 (54.42) | 2,420 (32.37) | 141,728 (46.19) |
|  | NQF6 (%) | 6,306 (12.00) | 12,949 (12.31) | 779 (10.42) | 37,249 (12.14) |
|  | NQF5 (%) | 2,831 (5.39) | 6,388 (6.07) | 294 (3.93) | 16,407 (5.35) |
|  | NQF4 (%) | 8,416 (16.02) | 17,173 (16.33) | 1,160 (15.52) | 54,693 (17.82) |
|  | NQF≤3 (%) | 9,062 (17.25) | 11,432 (10.87) | 2,823 (37.76) | 56,764 (18.50) |
|  | Unknown/Missing (%) | 876 (1.64) | 1,459 (1.37) | 177 (2.31) | 4,816 (1.55) |
| **Ever smoker^§^** | Yes (%) | 16,242 (30.41) | 27,773 (26.04) | 4,550 (59.45) | 94,930 (30.46) |
|  | Mean Pack Years (SD) | 24.27 (19.58) | 20.40 (16.85) | 38.24 (25.48) | 23.69 (18.42) |
|  | Unknown/Missing**^§^** | ≤ 2848 participants in total with “missing pack years” | | | |
| ^†^Participants stated they were diagnosed by a doctor; ^1^HAE: hay fever, allergic rhinitis, or eczema; ^2^COPD: chronic obstructive pulmonary disease (emphysema/chronic bronchitis); ^3^TDI: Townsend deprivation index; ^‡^According to the national qualifications framework in the UK; NQF7+: college or university degree + higher national degree/diploma, or national vocational qualification, NQF6: other professional qualification (e.g. nursing); NQF5: higher school certificate or equivalent (advanced (subsidiary) level); NQF4: lower school certificate or equivalent (ordinary level, (general) certificate of secondary education); NQF≤3: none of the above; ^§^Calculated from “pack years”. We display total participants with missing pack years data under “Unknown/Missing”. See text and UK Biobank Data-Fields 1239 & 2149 for further explanation. | | | | | |

**Table S2: Odds ratios & corresponding 95% intervals for 3rd trimester and 3 months post-birth photoperiod metric associations with COPD taking when smoking into account in the UK Biobank**

|  | **Mean daily photoperiod** | | | | **Relative photoperiod range** | | |
| --- | --- | --- | --- | --- | --- | --- | --- |
|  | **Model 2** | **Model 2 ns** | | **Model 2 s** | **Model 2** | **Model 2 ns** | **Model 2 s** |
| ***3^rd^ trimester*** | | | | | | | |
| **ESP** | 0.87 (0.81-0.94) | 0.95 (0.85-1.07) | | 0.84 (0.77-0.93) | 3.10 (1.82-5.26) | 1.22 (0.52-2.85) | 4.64 (2.35-9.15) |
| **NEP** | 1.02 (0.96-1.09) | 1.03 (0.93-1.13) | | 1.02 (0.94-1.11) | 2.66 (1.46-4.84) | 2.82 (1.14-7.00) | 2.14 (0.99-4.62) |
| **ELP** | 1.06 (0.97-1.17) | 0.95 (0.82-1.10) | | 1.10 (0.98-1.25) | 1.99 (0.83-4.75) | 0.65 (0.16-2.57) | 3.11 (1.00-9.70) |
| ***3 months post-birth*** | | | | | | | |
| **ESP** | 0.82 (0.76-0.88) | 0.88 (0.78-0.97) | | 0.80 (0.72-0.88) | 3.08 (1.78-5.29) | 1.75 (0.74-4.12) | 3.77 (1.86-7.64) |
| **NEP** | 1.07 (1.01-1.14) | 1.03 (0.94-1.12) | | 1.10 (1.01-1.19) | 3.42 (1.85-6.30) | 2.20 (0.91-5.32) | 3.75 (1.66-8.47) |
| **ELP** | 1.26 (1.03-1.23) | 0.97 (0.83-1.12) | | 1.18 (1.05-1.33) | 2.86 (1.23-6.66) | 0.70 (0.18-2.70) | 4.27 (1.43-12.7) |
|  |  |  | |  |  |  |  |
|  | **Model 2 s** | | **Model 2A** | | **Model 2 s** | **Model 2A** | **α_1_ × α_2_** |
| ***3^rd^ trimester*** | | | | | | | |
| **ESP** | 0.84 (0.77-0.93) | | 0.87 (0.79-0.96) | | 4.64 (2.35-9.15) | 3.45 (1.73-6.89) | 0.99 (0.98-1.01) |
| **NEP** | 1.02 (0.94-1.11) | | 1.02 (0.94-1.10) | | 2.14 (0.99-4.62) | 1.86 (0.86-4.01) | 1.03 (1.00-1.06) |
| **ELP** | 1.10 (0.98-1.25) | | 1.04 (0.92-1.18) | | 3.11 (1.00-9.70) | 1.74 (0.55-5.51) | 1.00 (0.99-1.02) |
| ***3 months post-birth*** | | | | | | | |
| **ESP** | 0.80 (0.72-0.88) | | 0.82 (0.74-0.91) | | 3.77 (1.86-7.64) | 3.11 (1.52-6.35) | 0.99 (0.98-1.01) |
| **NEP** | 1.10 (1.01-1.19) | | 1.04 (1.02-1.20) | | 3.75 (1.66-8.47) | 3.14 (1.41-6.98) | 1.03 (1.00-1.06) |
| **ELP** | 1.18 (1.05-1.33) | | 1.10 (0.97-1.24) | | 4.27 (1.43-12.72) | 2.15 (0.71-6.51) | 1.01 (1.00-1.03) |
| **Model 2:** photoperiod metrics, sex, ethnicity, age in total population  **Model 2 ns:** model 2 for “ever smoker - no” only  **Model 2 s:** model 2 for “ever smoker - yes” only  **Model 2A:** Model 2 s + pack years  **α_1_ × α_2_:** interaction term between pack years and relative photoperiod range  **Perinatal Photoperiod Metrics**  **Mean daily photoperiod** = average of photoperiods in the 3^rd^ trimester.  **Photoperiod relative range** = difference between longest and shortest photoperiods in the 3^rd^ trimester relative to the mean.  **Photoperiod Groups (by photoperiods experienced in the 3^rd^ trimester**  **ESP** = extreme short photoperiods (individuals who experience at least 1 day with photoperiod < 8 hours);  **NEP** = non-extreme photoperiod (individuals who experience 3^rd^ trimester photoperiods between 8 and 16 hours exclusively);  **ELP** = extreme long photoperiods (individuals who experience at least 1 day with photoperiod > 16 hours). | | | | | | | |

**References**

1. D’Elios M, Del Prete G. Th1/Th2 balance in human disease. *Transplant Proc*. 1998;30(5):2373-2377. doi:10.1016/S0041-1345(98)00659-9

2. Licona-Limón P, Kim LK, Palm NW, Flavell RA. TH2, allergy and group 2 innate lymphoid cells. *Nat Immunol*. 2013;14(6):536-542. doi:10.1038/ni.2617

3. García-Serna AM, Martín-Orozco E, Hernández-Caselles T, Morales E. Prenatal and perinatal environmental influences shaping the neonatal immune system: A focus on asthma and allergy origins. *Int J Environ Res Public Health*. 2021;18(8). doi:10.3390/ijerph18083962

4. Jiang M, Tao S, Zhang S, et al. Type 2 innate lymphoid cells participate in IL‑33‑stimulated Th2‑associated immune response in chronic obstructive pulmonary disease. *Exp Ther Med*. Published online August 20, 2019. doi:10.3892/etm.2019.7924

5. Wilczyński JR. Th1/Th2 cytokines balance—yin and yang of reproductive immunology. *European Journal of Obstetrics & Gynecology and Reproductive Biology*. 2005;122(2):136-143. doi:10.1016/J.EJOGRB.2005.03.008

6. Dietert RR. Developmental immunotoxicology (DIT): Windows of vulnerability, immune dysfunction and safety assessment. *J Immunotoxicol*. 2008;5(4):401-412. doi:10.1080/15476910802483324

7. Lehmann I, Thoelke A, Weiss M, et al. T cell reactivity in neonates from an East and a West German city - Results of the LISA study. *Allergy: European Journal of Allergy and Clinical Immunology*. 2002;57(2):129-136. doi:10.1046/j.0105-4538.2002.00001.x

8. Sullivan Dillie KT, Tisler CJ, Dasilva DF, et al. The influence of processing factors and non-atopy-related maternal and neonate characteristics on yield and cytokine responses of cord blood mononuclear cells. *Clinical and Experimental Allergy*. 2008;38(2):298-304. doi:10.1111/j.1365-2222.2007.02891.x

9. Coomans CP, Ramkisoensing A, Meijer JH. The suprachiasmatic nuclei as a seasonal clock. *Front Neuroendocrinol*. 2015;37:29-42. doi:10.1016/J.YFRNE.2014.11.002

10. Ehlers A, Xie W, Agapov E, et al. BMAL1 links the circadian clock to viral airway pathology and asthma phenotypes. *Mucosal Immunol*. 2018;11(1):97-111. doi:10.1038/MI.2017.24

11. Akashi M, Takumi T. The orphan nuclear receptor RORα regulates circadian transcription of the mammalian core-clock Bmal1. *Nat Struct Mol Biol*. 2005;12(5):441-448. doi:10.1038/nsmb925

12. Kim HY, Umetsu DT, Dekruyff RH. Innate lymphoid cells in asthma: Will they take your breath away? *Eur J Immunol*. 2016;46(4):795-806. doi:10.1002/eji.201444557

13. Halim TYF, MacLaren A, Romanish MT, Gold MJ, McNagny KM, Takei F. Retinoic-Acid-Receptor-Related Orphan Nuclear Receptor Alpha Is Required for Natural Helper Cell Development and Allergic Inflammation. *Immunity*. 2012;37(3):463-474. doi:10.1016/j.immuni.2012.06.012

14. Lee JE, Choi G, Cho M, Kim D, Lee MO, Chung Y. A critical regulation of Th2 cell responses by RORα in allergic asthma. *Sci China Life Sci*. 2021;64(8):1326-1335. doi:10.1007/s11427-020-1825-3

15. Orihara K, Haraguchi A, Shibata S. Crosstalk among circadian rhythm, obesity and allergy. *Int J Mol Sci*. 2020;21(5). doi:10.3390/ijms21051884

16. Hahn K, Sundar IK. Current Perspective on the Role of the Circadian Clock and Extracellular Matrix in Chronic Lung Diseases. *Int J Environ Res Public Health*. 2023;20(3). doi:10.3390/ijerph20032455

17. Li L, Zhang M, Zhao C, Cheng Y, Liu C, Shi M. Circadian clock gene Clock-Bmal1 regulates cellular senescence in Chronic obstructive pulmonary disease. *BMC Pulm Med*. 2022;22(1). doi:10.1186/s12890-022-02237-y

18. Kadalayil L, Alam MZ, White CH, et al. Analysis of DNA methylation at birth and in childhood reveals changes associated with season of birth and latitude. *Clin Epigenetics*. 2023;15(1). doi:10.1186/s13148-023-01542-5

19. Palomino-Segura M, Hidalgo A. Circadian immune circuits. *Journal of Experimental Medicine*. 2021;218(2). doi:10.1084/JEM.20200798

20. Shin YH, Hwang J, Kwon R, et al. Global, regional, and national burden of allergic disorders and their risk factors in 204 countries and territories, from 1990 to 2019: A systematic analysis for the Global Burden of Disease Study 2019. *Allergy: European Journal of Allergy and Clinical Immunology*. 2023;78(8):2232-2254. doi:10.1111/all.15807

21. Husna Tan H. Shukri N. Ashari N. Wong K. S. Allergic Rhinitis: A Clinical and Pathophysiological Overview. *Sec Pulmonary Medicine*. 2022;9-2022. doi:https://doi.org/10.3389/fmed.2022.874114

22. Song P, Adeloye D, Salim H, et al. Global, regional, and national prevalence of asthma in 2019: A systematic analysis and modelling study. *J Glob Health*. 2022;12. doi:10.7189/JOGH.12.04052

23. Adeloye D, Song P, Zhu Y, Campbell H, Sheikh A, Rudan I. Global, regional, and national prevalence of, and risk factors for, chronic obstructive pulmonary disease (COPD) in 2019: a systematic review and modelling analysis. *Lancet Respir Med*. 2022;10(5):447-458. doi:10.1016/S2213-2600(21)00511-7

24. Zuberbier T, Lötvall J, Simoens S, Subramanian S V., Church MK. Economic burden of inadequate management of allergic diseases in the European Union: A GA2LEN review. *Allergy: European Journal of Allergy and Clinical Immunology*. 2014;69(10):1275-1279. doi:10.1111/all.12470

25. Portaluppi F, Smolensky MH, Touitou Y. ETHICS AND METHODS FOR BIOLOGICAL RHYTHM RESEARCH ON ANIMALS AND HUMAN BEINGS. *Chronobiol Int*. 2010;27(9-10):1911-1929. doi:10.3109/07420528.2010.516381

26. Allen NE, Sudlow C, Peakman T, Collins R. UK biobank data: Come and get it. *Sci Transl Med*. 2014;6(224). doi:10.1126/scitranslmed.3008601

27. Palmer LJ. UK Biobank: bank on it. *The Lancet*. 2007;369(9578):1980-1982. doi:10.1016/S0140-6736(07)60924-6

28. Fry A, Littlejohns TJ, Sudlow C, et al. Comparison of Sociodemographic and Health-Related Characteristics of UK Biobank Participants with Those of the General Population. *Am J Epidemiol*. 2017;186(9):1026-1034. doi:10.1093/aje/kwx246

29. Scadding GK, Kariyawasam HH, Scadding G, et al. BSACI guideline for the diagnosis and management of allergic and non-allergic rhinitis (Revised Edition 2017; First edition 2007). *Clinical and Experimental Allergy*. 2017;47(7):856-889. doi:10.1111/cea.12953

30. British Lung Foundation. Chronic obstructive pulmonary disease (COPD) statistics. March 28, 2024. Accessed March 28, 2024. https://statistics.blf.org.uk/copd

31. Scholes S, Mindell JS. *Health Survey for England 2018 Asthma*.; 2019.

32. Lewis P, Morfeld P, Mohren J, Hellmich M, Erren TC. Perinatal photoperiod associations with diabetes and chronotype prevalence in a cross-sectional study of the UK Biobank. *Chronobiol Int*. 2021;38(3):343-359. doi:10.1080/07420528.2020.1849254

33. Lewis P, Gottlieb JF, Morfeld P, Hellmich M, Erren TC. Perinatal photoperiod associations with bipolar disorder and depression: A systematic literature review and cross-sectional analysis of the UK Biobank database. *Psychiatry Res*. Published online March 2024:115878. doi:10.1016/j.psychres.2024.115878

34. Haldar P, Carsin AE, Debnath S, et al. Individual circadian preference (Chronotype) is associated with asthma and allergic symptoms among adolescents. *ERJ Open Res*. 2020;6(2):1-4. doi:10.1183/23120541.00226-2020

35. Han CH, Chung J. Late chronotype is associated with adolescent asthma: Assessment using the Korean-version MCTQ. *Int J Environ Res Public Health*. 2020;17(9). doi:10.3390/ijerph17093000

36. Basnet S, Merikanto I, Lahti T, et al. Associations of common noncommunicable medical conditions and chronic diseases with chronotype in a population-based health examination study. *Chronobiol Int*. 2017;34(4):462-470. doi:10.1080/07420528.2017.1295050

37. Deprato A, Maidstone R, Cros AP, et al. Influence of light at night on allergic diseases: a systematic review and meta-analysis. *BMC Med*. 2024;22(1). doi:10.1186/s12916-024-03291-5

38. Raboso Moreno B, López Riolobos C, Díaz-García JM, Matesanz López C, Abad Fernández A. Influence of Chronotypes in Respiratory Pathology. *Open Respiratory Archives*. 2023;5(1). doi:10.1016/j.opresp.2022.100228

39. Erren TC, Morfeld P. Circadian epidemiology: Structuring circadian causes of disease and practical implications. *Chronobiol Int*. 2024;41(1):38-52. doi:10.1080/07420528.2023.2288219

40. Rothman KJ. Significance Questing. *Ann Intern Med*. 1986;105(3):445. doi:10.7326/0003-4819-105-3-445

41. Buckles KS, Hungerman DM. Season of birth and later outcomes: Old questions, new answers. *Review of Economics and Statistics*. 2013;95(3):711-724. doi:10.1162/REST_a_00314
